# Investigating Neuromagnetic Brain Responses against Chromatic Flickering Stimuli by Wavelet Entropies

**DOI:** 10.1371/journal.pone.0007173

**Published:** 2009-09-25

**Authors:** Mayank Bhagat, Chitresh Bhushan, Goutam Saha, Shinsuke Shimjo, Katsumi Watanabe, Joydeep Bhattacharya

**Affiliations:** 1 Department of Electrical Engineering, Indian Institute of Technology, Kharagpur, India; 2 Electronics & Electrical Communication Engineering, Indian Institute of Technology, Kharagpur, India; 3 Department of Biology, California Institute of Technology, Pasadena, California, United States of America; 4 NTT Communication Sciences Laboratories, Atsugi, Kanagawa, Japan; 5 Exploratory Research for Advanced Technology (ERATO), Japan Science & Technology Agency, Atsugi, Kanagawa, Japan; 6 Research Center of Advanced Science and Technology, University of Tokyo, Tokyo, Japan; 7 National Institute of Advanced Industrial Science and Technology, Tsukuba, Ibaraki, Japan; 8 Department of Psychology, Goldsmiths College, University of London, London, United Kingdom; 9 Commission for Scientific Visualization, Austrian Academy of Sciences, Vienna, Austria; Tel Aviv University, Israel

## Abstract

**Background:**

Photosensitive epilepsy is a type of reflexive epilepsy triggered by various visual stimuli including colourful ones. Despite the ubiquitous presence of colorful displays, brain responses against different colour combinations are not properly studied.

**Methodology/Principal Findings:**

Here, we studied the photosensitivity of the human brain against three types of chromatic flickering stimuli by recording neuromagnetic brain responses (magnetoencephalogram, MEG) from nine adult controls, an unmedicated patient, a medicated patient, and two controls age-matched with patients. Dynamical complexities of MEG signals were investigated by a family of wavelet entropies. Wavelet entropy is a newly proposed measure to characterize large scale brain responses, which quantifies the degree of order/disorder associated with a multi-frequency signal response. In particular, we found that as compared to the unmedicated patient, controls showed significantly larger wavelet entropy values. We also found that Renyi entropy is the most powerful feature for the participant classification. Finally, we also demonstrated the effect of combinational chromatic sensitivity on the underlying order/disorder in MEG signals.

**Conclusions/Significance:**

Our results suggest that when perturbed by potentially epileptic-triggering stimulus, healthy human brain manages to maintain a non-deterministic, possibly nonlinear state, with high degree of disorder, but an epileptic brain represents a highly ordered state which making it prone to hyper-excitation. Further, certain colour combination was found to be more threatening than other combinations.

## Introduction

Photosensitivity has been a topic of interest in medical field during last six decades [1], [2], [3], [4], [5], [6], [7], [8]. Studies show that visual flicker stimulus induces bilateral rhythmic activity in brain which is harmonically related to the flicker frequency itself [7] This phenomenon known as photic driving manifests itself through photomyogenic (clonic spasm of muscles) and photoparoxysmal (spike-wave complexes in brain activities) responses in humans while being stimulated by flickering visual stimulus [2], [9]. While the former is found in elderly populations, the latter is found predominantly in children and in adolescents [4], [7]. Flicker-evoked paroxysmal epileptic discharges, characterized by spikes, poly-spikes, and spike-wave complexes in Electroencephalography (EEG) recording, indicate Photosensitive Epilepsy (PSE) [4], [6], [10], [11], [12].

The issue of PSE has recently shot to public domain limelight in December 1997, when a television program “*Pokemon*” triggered concurrent appearance of convulsive response in about 700 individuals, causing a nation wide panic, in Japan [5], [13]. The acute symptoms included convulsive fits and loss of consciousness in children and young individuals, who were exposed to flickering stimulus during the televised program. The range of stimuli which can potentially trigger PSE varies from natural sunlight flickers between trees to artificial illumination of discotheques [14]. Television remains most trite stimulus in this case [1]. Some cases were also reported with pattern flickering such as that of black and white stripes and bars on the stairs of escalators [4]. However, amongst all possible parameters of a visual stimuli, chromaticity is less studied in the context of PSE.

Studies of electrophysiological signals noninvasively obtained from brains with neurological disorders may give an insight into the temporal and spatial effects of the disorders [15], [16], [17], [18], [19], [20], [21], [22]. EEG stands out as the most widely studied physiological signal for investigating photosensitive epilepsy [17], [23], [24]. However, recently magnetoencephalography (MEG) has also gained importance as a noninvasive novel technique for investigating photosensitivity [25], [26]. Both EEG and MEG are generated by synchronous oscillation of pyramidal neurons, but MEG may have some innate advantages over EEG [27], [28], [29] (but also see [30]). For example, unlike EEG, MEG is free of reference problem, i.e. it does not require any reference in the measurement, and is less prone to the heterogeneities of the skull which often distorts the EEG signals recorded from the scalp. The research on photosensitivity using MEG signals has been focused predominantly on the features based on nonlinear dynamical system theory [31]. This paper presents a feature based analysis of photosensitivity with MEG signals being dealt in discrete wavelet domain.

Classically, Fourier analysis decomposes a signal into sine/cosine waves of various frequencies [32]. On the other hand, wavelet analysis decomposes a signal into shifted and scaled versions of the original (or mother) wavelet [33]. Therefore, wavelet has an intrinsic attribute of analyzing signal at different frequencies with different resolutions [34], making it a powerful alternative to the Fourier method for the time-frequency analysis of non-stationary signals like EEG [35], [36], [37], [38], [39], [40], [41], [42]. Feature based analysis of wavelet decomposed signal requires identification of representative measures of the signal. Wavelet entropy is a recently proposed nonparametric measure to characterize large scale brain responses [43]. Wavelet entropy represents a measure of the degree of order-disorder associated with a multi-frequency signal response, or in other terms, reflects a degree of uncertainty of spectral power distribution of a signal in different wavelet bands [44], [45]. In this study, we applied a family of wavelet entropies which are based on Shannon entropy formulation as well as on different entropy-forms (i.e. Renyi entropy, Tsallis entropy) [35] to the MEG signals obtained from control/healthy participants and from patients, both unmedicated and medicated with sodium valporate (an antiepileptic drug), suffering from photosensitive epilepsy while they were being visually stimulated by three types of chromatic flickering stimuli.

The prime objectives of this study were three-folds: (i) to differentiate between controls and patients, however indicative or suggestive, (ii) to study the spatio-temporal dynamics of brain order/disorder when stimulated by flickering stimuli, and finally (iii) to study the sensitivity against different color combinations. Considering the epilepsy as a dynamical disease of brain system [46], [47], we predicted that the unmedicated patient would be associated with lower degree of entropy as compared to controls. We also predicted that medicated patient would be closer to the controls than to the unmedicated patient. Finally, based on our earlier results [48], [49] we expected that Red/Blue flickering stimulus would elicit the most robust changes in wavelet entropy.

## Materials and Methods

All adult participants (and children's parents when the participants were children) gave their written informed consent. Experimental protocols, set according to the Helsinki Declaration, were approved by the Internal Review Board of the National Children's Hospital, Japan. A pediatric neurologist, who continuously monitored MEG activity online, and the parents of the children participating in the study observed the experiment. The experiment was to be terminated if either the physicians or the parents suspected the child's safety was at risk, or if the child felt ill, but no such incident occurred.

### Participants

The participants belonged to four categories: adult healthy controls, an unmedicated patient, age-matched controls, and a medicated patient. There were nine healthy/control participants (seven males) with an age range of 22–27 years. It was ensured that none of the control participants had any kind of personal or family history of PSE. The patient was a 12 year old female photosensitive patient who was without any medication. Two additional female participants age-matched with the patient were considered as age-matched controls. Finally, we also studied a medicated female patient, who was earlier diagnosed with photosensitive epilepsy but under medication of sodium valporate, an antiepileptic drug.

### Stimuli

Visual stimuli, which may trigger PSE, are characterized by myriads of visual parameters like temporal frequency, spatial frequency, stimulus contrast, patterns, and chromaticity. This makes the choice of visual stimuli a critical decision depending on the aspect of PSE on which one would like to focus. Of all visual parameters, chromaticity is less studied, yet its importance in the age of multimedia with numerous colour displays can hardly be overstated. Therefore, in our study, we used three types of flickering stimuli: Red/Blue, Red/Green, and Blue/Green. In our study, visual stimuli were generated by using two video projectors. Each of these projectors produced a single color stimulus, and a LCD shutter was placed between the optical device and each projector. The chromatic flicker was produced by alternate opening and closing of the two LCD shutters, and the temporal frequency of the stimulus was 10 Hz. This chromatic flicker was then projected on to a viewing screen, placed 30 cm from the participants. Isoluminant stimuli for three color combinations Red/Blue, Red/Green, and Blue/Green, were generated with an objective luminosity of 1.6 cd/m^2^. The color contrast chosen emulate the parameters in typical color television monitors. The CIE chromaticity coordinates were (0.496, 0.396) for red, (0.153, 0.122) for blue, and (0.308, 0.522) for green.

### Data acquisition and preprocessing

**Figure 1:**
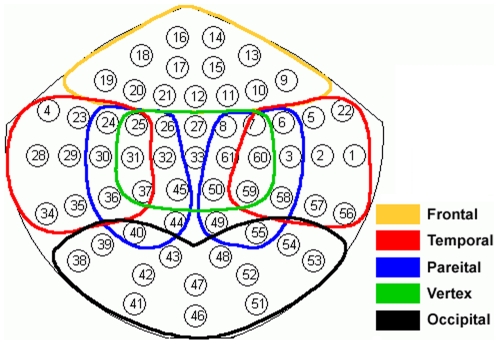
Sensor locations for full scalp MEG. Scalp was divided into seven regions, frontal, left temporal, right temporal, left parietal, right parietal, vertex, and occipital, respectively.

Visually evoked neuromagnetic responses of the brain were recorded by a whole scalp MEG system. The 122 channel and 61 sensor instrument (Neuromag-122, Neuromag Ltd, Finland) had two orthogonally oriented planar gradiometers at each of 61 sensor location, coupled to dc-SQUID sensors. Fig. 1 shows the spatial locations of the sensors. For extended comprehensibility, Fig. 1 also shows the division of sensors in seven cortical regions as done earlier [31]. This division contains 13 sensors in the frontal region, 14 in the vertex and occipital, 12 in the left and right temporal, and 11 in the left and right parietal region. Due to overlap between regions, a sensor may belong to more than one region.

During each trial the participants were exposed to the flickering stimulus for 3 s (2 s for patients) with a gap of 3 s between consecutive trials. The prestimulus baseline was 200 ms prior to the application of visual stimulus. The MEG signals were band-pass filtered between 0.03 and 100 Hz and sampled at a rate of 500 Hz. Eye blink and eye movements were monitored by recording EOG signals, and trials containing eye-artefacts were eliminated from further processing. For each chromatic flickering and at each sensor, more than 80 artefact free trials were obtained and subsequently averaged to generate the event-related-field, which was further band-pass filtered between 0.5 Hz and 40 Hz to produce the final data set.

### Discrete Wavelet Transform (DWT)

Since the adopted family of wavelet entropy crucially depends on the concept of discrete wavelet transform (DWT), we described here some key details of DWT. Unlike the Fourier transform which is precisely localized in frequency domain but infinitely extended in time domain, wavelet transform offers a good localization in both time and frequency domains. Therefore, the wavelet transform not only captures the neuronal transients but also provides information about the temporal evolution of the constituent frequency components.

Any finite energy time domain signal can be represented as summation of a family of ‘baby wavelets’ 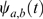, which are scaled (by stretching or shrinking) and translated (moving to different time positions) versions of a mother wavelet 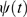 which are transformed to locally fit in the original signal. The family of baby wavelets can be derived from mother wavelet by
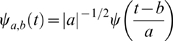
(1)where 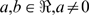 are scale and translation parameters, respectively, and *t* is time.

In DWT, the values of 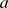 and 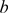 are restricted to discrete multiple of two, given as 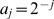 and 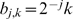, where 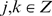 (Z being the set of integers) represent levels of resolution and sampled time, respectively. Therefore, the wavelet family can be given as
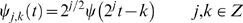
(2)


Discrete wavelet coefficients *C_j_*(*k*) are defined as the correlations of a given signal *S*(*t*) with wavelet function 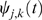. The biggest attribute of DWT is its ability to analyze a signal using multi-resolution schemes in 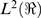 (set of all energy signals). Assuming a hypothetical case of splitting a signal to infinite resolution levels, we can define a signal in terms of its discrete wavelet coefficients, as follows
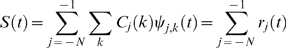
(3)where,
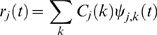
(4)is the *detailed signal* at scale *j*, and *provides* information localized in the frequency band 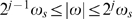 where 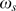 is the sampling frequency.

The choice of mother wavelet plays a critical role in extraction of the desired features from the signal. A rule of thumb is to select the method wavelet that is similar to the waveform of the original signal. In this study, owing to the spiky shape of the MEG, coiflet-4 has been chosen as the mother wavelet.

Since the neural data are often nonstationary [50], [51], we divided the signal for each trial (3 s for controls and 2 s for patient) into smaller (400 ms) but relatively stationary windows, and two successive windows had a overlap of 200 ms. All successive computations were done with this windowing scheme.

### Shannon based wavelet Entropy (SE)

Wavelet entropy, a measure of the order/disorder of signal, quantifies the probability of occurrence of particular oscillatory component. A signal having higher probability of occurrence has lower disorder and hence lower entropy. SE was calculated as follows.

First, the total energy content of the *i*-th window resolved into *j* levels was calculated directly from its discrete wavelet coefficients as
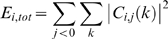
(5)


The total energy at a particular level *j* in the given *i*-th window can be expressed as
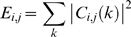
(6)


The probability distribution of the energies present in each resolution level *j* is given by
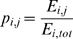
(7)


The earlier term (7) is known as relative wavelet energy and its distribution is considered as a time-scale empirical probability density function suitable for characterization of signals on time-scale (or equivalently on time-frequency) domains [44]. For possible comparison between different probability distributions, Shannon entropy (SE) is calculated,
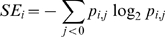
(8)


**Figure 2:**
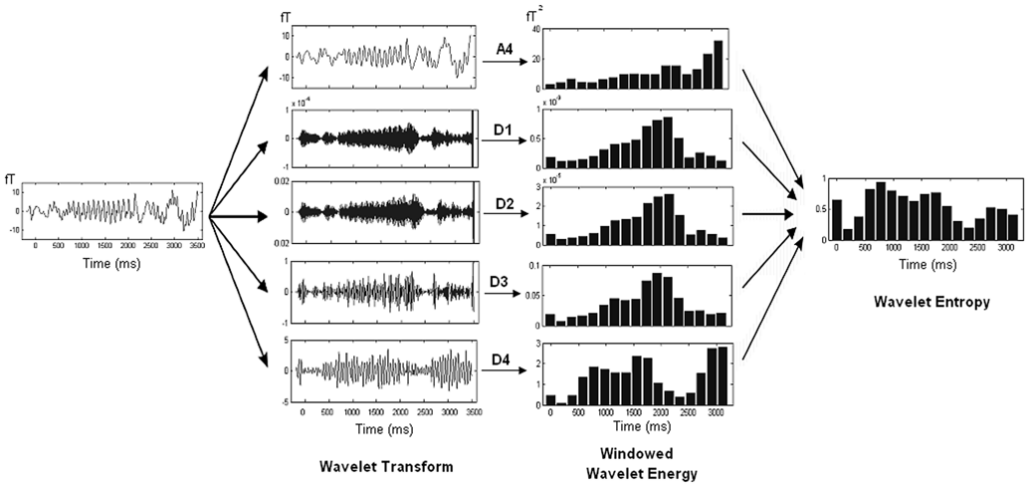
Systematic illustration of wavelet entropy method. MEG signal was first transformed to mutiresolution time-frequency domain by wavelet transformation. Then the values were windowed and corresponding energy was computed in all resolution. Finally wavelet entropy was computed.

The above term is defined as Shannon wavelet entropy and provides a probabilistic measure of order/disorder in the signal. A signal having very high energy content in a particular resolution level *j* underscores the fact that it is predominantly composed of particular frequency band. The concentration of energy in a particular frequency band indicates lack of randomness in terms of frequency of that particular signal. Hence the entropy value will be lower for such signals. On the other hand, uniform distributions of energy across levels suggests a presence of randomness in the signal, thus leading to higher entropy values. Fig. 2 illustrates the scheme to compute the wavelet entropy.

### Other Entropy Measures

Though Shannon entropy based quantifier has been quite successful, we also applied other related measures as follows. The first one computed is known as Rényi entropy (RE), which can be conceptualized as superclass of Shannon entropy [37], and is given by
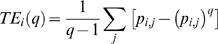
(9)where *p_i,j_* is the probability function as described earlier and 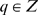 is known as entropic index. The parameter 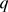 generalizes the information measure. As *q* approaches 1, *RE* converges to *SE*. In the present study, we used *q* = 2, 3 to calculate 2^nd^ and 3^rd^ order Renyi entropy, which were termed as RE2 and RE3, respectively.

Both *RE* and *SE* represent an extensive property of a system, where the extensive nature is manifested by the additive attribute of the property.

Next we computed nonextensive entropies like Tsallis wavelet entropy (TE) [52] and generalized escort-Tsallis wavelet entropy (*GE*) [53]. Being non-extensive in nature these entropies are governed by pseudo-additive theorems instead of by additive ones. The major difference between extensive and non-extensive entropy is that the former is suitable for signals with short-range interactions whereas the latter is suitable for signals with long-range interactions. TE is a non-logarithmic parameterized entropy defined as [52]

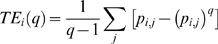
(9)where 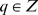 is a degree of non-extensivity. Low values of *q* are appropriate for signals having long range interactions, whereas higher values of 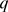 are used with signals plagued with spikes and sudden abrupt changes. In this study we have used *q* = 2 for TE.

*GE* is defined as [53]

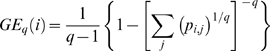
(10)where *q* is the entropy parameter similar to that used for TE. *GE* shares its non-extensive properties with TE but differs in its treatment of probability distributions. The probability distribution is modified to generate an escort distribution of order *q*. The *q* value for this study was taken to be two.

These various kinds of wavelet entropies constitute a wide range of features that we used in this study in order to obtain a comprehensive insight into the neuronal oscillatory dynamics of PSE.

## Results and Discussion

The MEG signals of this study came from nine healthy adult human participants as controls, one unmedicated patient diagnosed with photosensitive epilepsy, one medicated patient, and two more controls age-matched with the patient. For a comprehensive insight into the underlying brain mechanism, we divided the result section into individual subsections as follows: temporal analysis, spatial analysis, entropic variations, spatio-temporal analysis, chromatic sensitivity, and effect of medication.

### Temporal analysis

**Figure 3:**
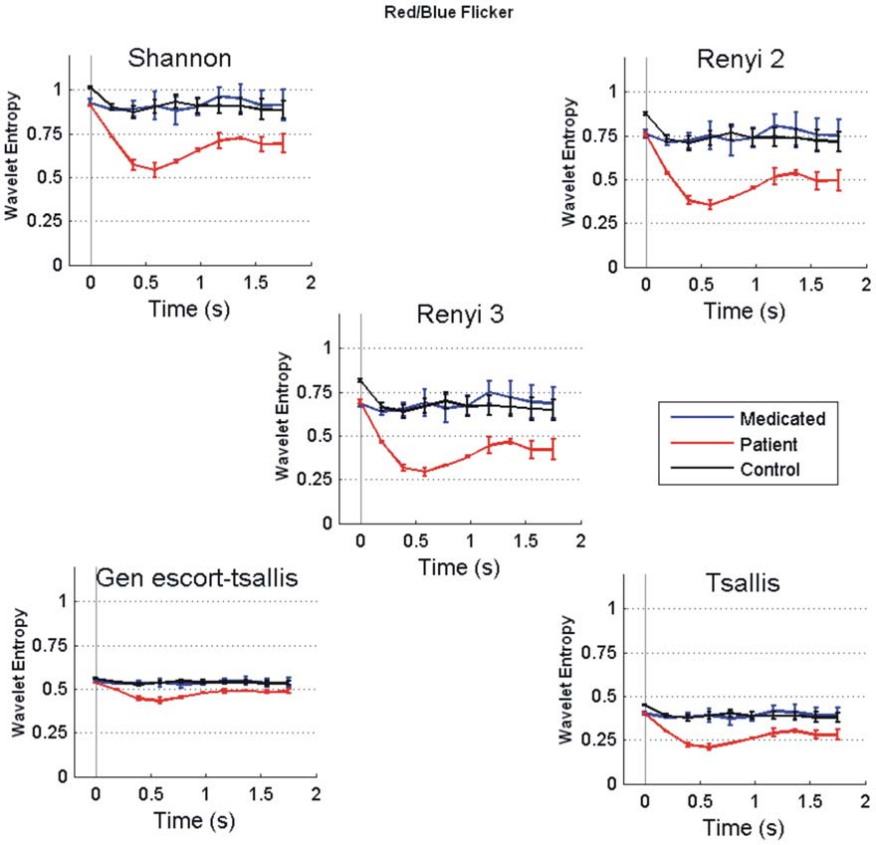
Temporal analysis. Temporal evolution of entropy values (mean±s.e.) for Red/Blue flicker. Results were first averaged over all the sensors for each participant. Each point represents the center of each window which was 400 ms long. The onset of visual stimulus was indicated by a vertical line. Note similar entropy values for all the participants at the first window ranging from 200 ms prestimulus to 200 ms post-stimulus. However, entropy values reduced drastically in the patient at later stages of poststimulus processing.

**Figure 4:**
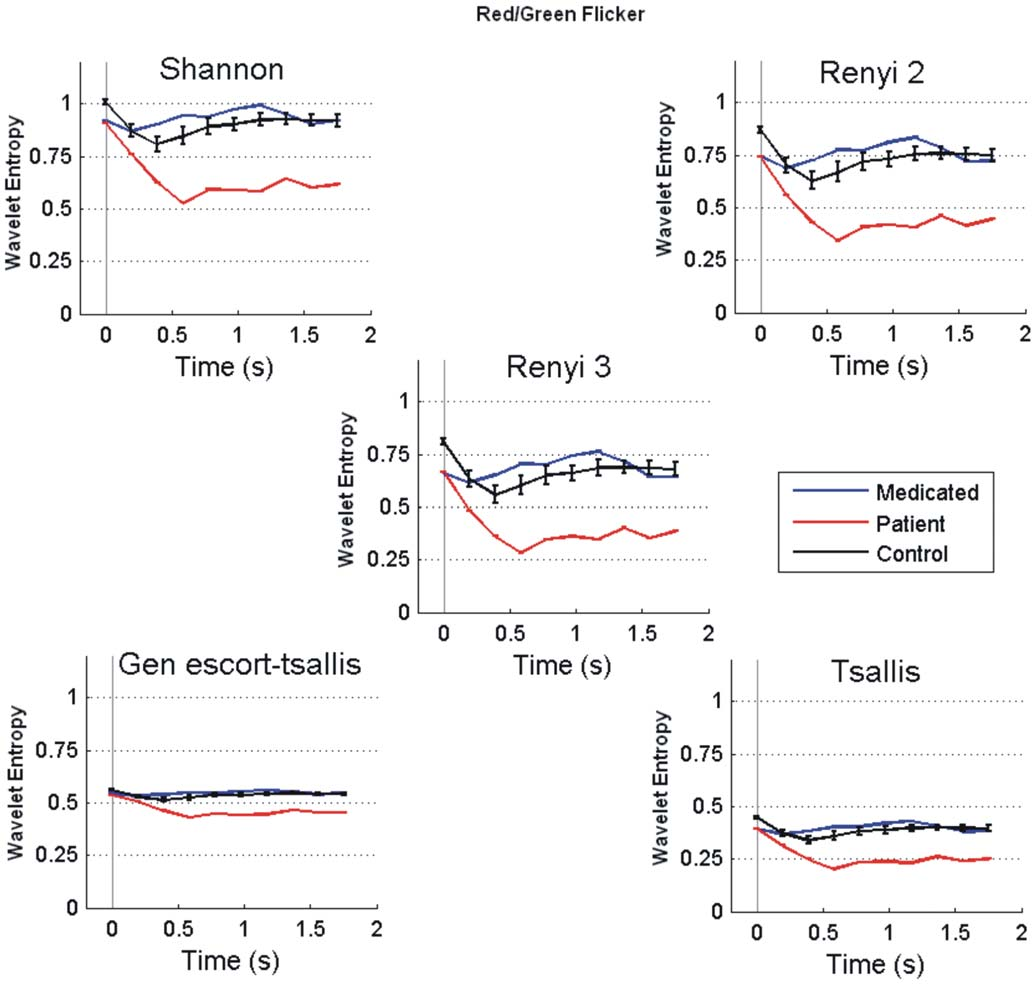
Temporal analysis. Same as Fig. 3 but for Red/Green flickering.

The temporal evolutions of five different types of wavelet entropies, averaged across all sensors, for Red/Blue flickering stimulus for controls, unmedicated and medicated patients are shown in Fig. 3. Following noteworthy features could be noted which were invariant across different types of entropies. The entropy value at the first window, which ranges from −200 ms to 200 ms (0 ms representing the stimulus onset), was similar across controls and the patients. However, the differences between controls and the unmedicated patient got obvious from the second window which ranges from 0–400 ms. Second window onwards the entropy value for all categories dropped from their individual baselines. This reduction of entropy values strengthens the hypothesis regarding the transformation of brain from a highly disordered nonlinear system to a more ordered one after the onset of stimulus processing. However, this fall was rather small in controls and in medicated patient, suggesting their resilience to the flickering stimulus. But this effect was much more pronounced in the unmedicated patient suffering from PSE, indicating her vulnerability to get entrained by the flickering stimulus which could eventually lead to a cortical hyper-excitation. At later time period, we found that the unmedicated patient's profile was totally separated from controls and the medicated patient: in subsequent time-windows (400–2000 ms) entropy values of unmedicated patient were significantly lower than those of others. Interestingly, at the end of each trial controls and medicated patient almost regained their baseline values but for the unmedicated patient it remained at a much lower value. The temporal variation of the feature expressed as mean of various individuals within the same class also shows the error bar at each time window symbolizing the range in which individual values may lie for a certain subject class. In the case of Red/Blue flicker (Fig. 3) we have taken two trials each for medicated and unmedicated patient, hence the error bar shows the variation in these two trials. Though the above analysis was based on Red/Blue flicker, similar differences between controls and patients were obtained for Red/Green (Fig. 4) and Blue/Green flickering stimulus (figure not shown). This simple analysis based on wavelet entropy fulfils one of our fundamental objectives of separating unmedicated patient from the rest in a robust manner.

### Spatial analysis

**Figure 5:**
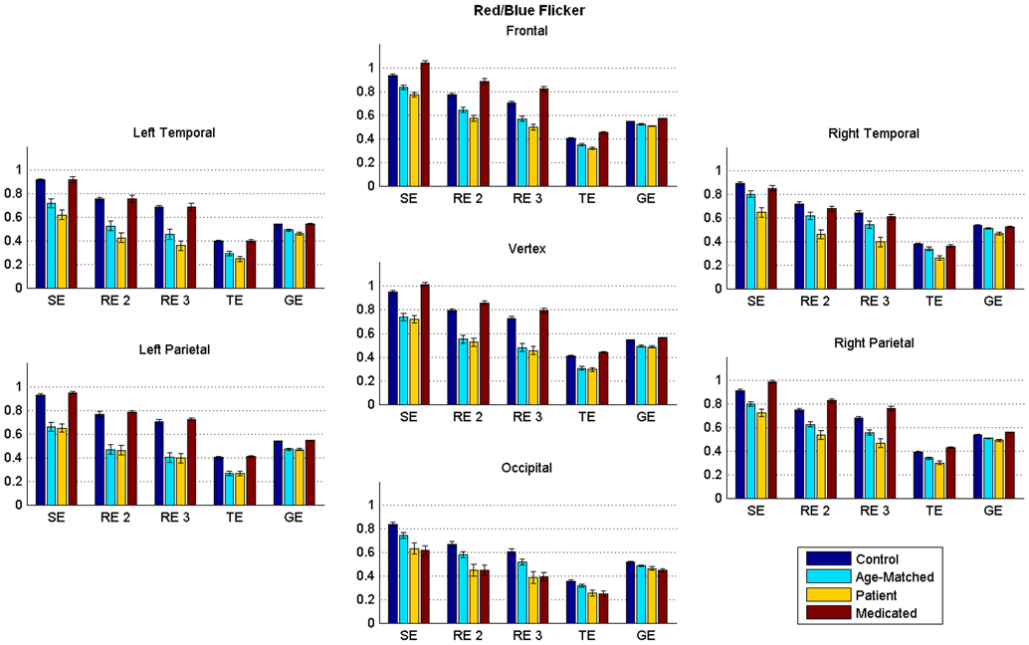
Spatial analysis. Entropy values (mean±s.e.) in seven broad cortical regions for Red/Blue flickering stimulus for adult controls, one unmedicated patient, one medicated patient, and two further controls age-matched with patients. Then mean entropy value across time windows excluding the first window was calculated within each category. Note lower entropy values for unmedicated patients and higher entropy values for control participants across cortical regions.

For spatial analysis, we divided the brain into seven cortical regions (Fig. 1) and averaged the entropy values across sensors within each region. In the next stage of data reduction, the mean entropy across different time windows excluding the first time window (post-stimulus part only) was computed and Fig. 5 shows its variations in seven cortical regions during Red/Blue condition. Further to show the variability of entropy values in subsequent time windows, we calculate standard error across the entire post-stimulus time windows. This is represented as error bar over mean in Fig. 5. In each cortical region five clusters of bars represent five different types of entropies. According to our hypothesis a high mean value for controls and low mean value for unmedicated patient were expected. Typically, the frontal region showed high values of entropy in both controls and in medicated patient, while the lowest entropy value was seen in patient, and the entropy value for age matched controls was in between the controls and the unmedicated patient. Similar trends were found in the temporal regions, bilaterally, but not in the occipital region, where the entropy values for medicated patient and unmedicated patient were comparable. This latter effect is explained later. Altogether, similar results were observed for Red/Green and Blue/Green flickering stimuli.

**Figure 6:**
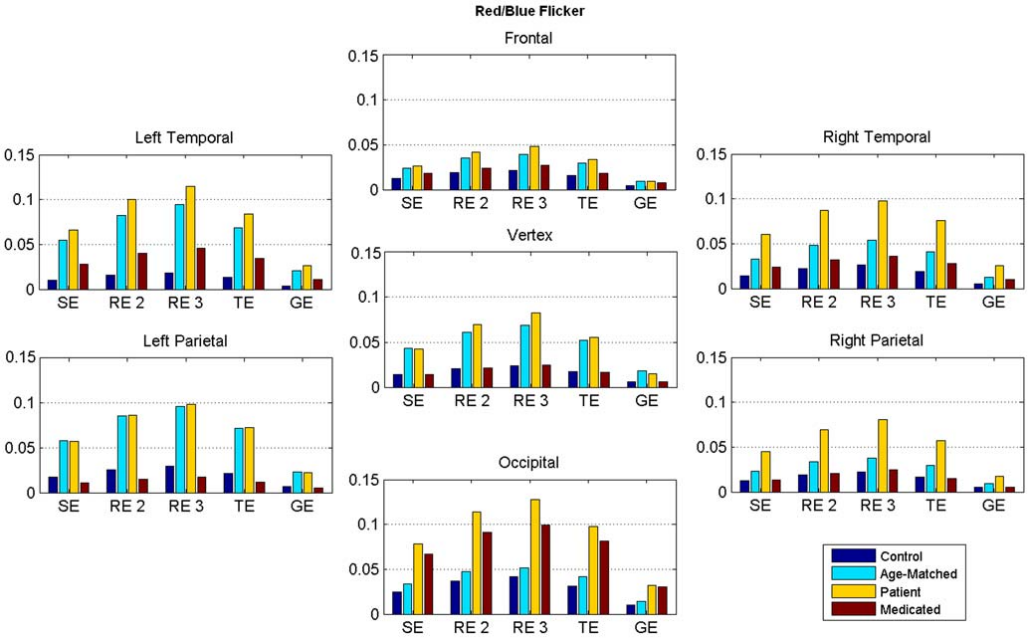
Coefficient of variation. Ratio of standard error to mean for all entropies in seven cortical regions for Red/Blue Flicker, Note higher values of this ratio in patients and lower values in controls at different regions.

In order to obtain further insight into the temporal dynamics of individual cortical region, we used a statistical measure called the coefficient of variation defined as the ratio of standard error to mean of the entropy values computed over successive time windows. Our earlier temporal analysis (Fig. 3) showed a trend of large deviation and low mean in unmedicated patient and small deviation and high mean in controls. Fig. 6 shows the values of this ratio for all five types of entropies and at seven different cortical regions. As expected, the unmedicated patient produced the highest ratio, and the adult controls the lowest, whereas the medicated patient was somewhere between these two categories. Further, the highest values were observed at the occipital region, which might be crucially involved with photosensitivity since occipital cortex is where the visual stimulus is predominantly processed. Interestingly, the medicated patient exhibited a behavior quite similar to control participants in other cortical regions except the occipital. This might suggest that the medicated patient was more vulnerable than controls in processing flickering stimulus. This simple index may help in designing a bio-marker by which one could differentiate a patient from a healthy participant by setting a threshold value for this index. Any value higher than this threshold would therefore potentially indicate the category membership. However, further research with a larger pool of control participants and of patients are needed to be tested beforehand.

### Entropy comparison

An interesting aspect would be to compare the performances of different entropy measures. Although different entropies produced similar results in terms of temporal and spatial profiles, subtle variations also did exist. For example, the results based on GE of 2^nd^ order produced less dramatic differences between controls and unmedicated patient (Figs. 3, 5). Next in the increasing order of contrast comes the classical SE for which its value was higher than GE, but was lower than the rest. Moving along the same trend of increasing contrast we found that TE and RE2 provided better results as compared to SE and GE. But RE3 has emerged to be the best in terms of performance. It has a characteristic high value in unmedicated patient and offered very well formulated differences between different participants, thus RE3 could be considered as a powerful feature for participant classification.

### Spatiotemporal analysis

**Figure 7:**
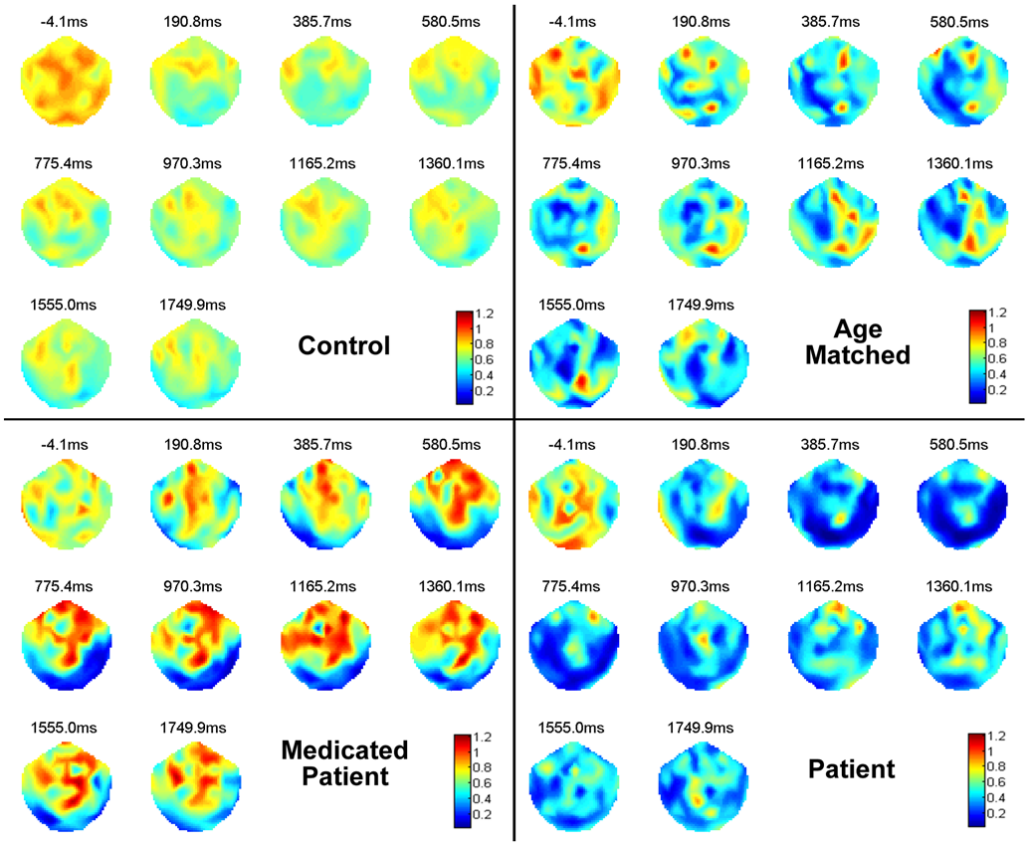
Spatiotemporal progression of wavelet entropy. Values of RE3 were plotted over successive time windows for participants belonging to the four categories. Note similar entropy distributions across sensors at the very beginning across all participants but differences became conspicuous at later stages.

Fig. 7 shows the topographical maps of RE3 values over successive time windows for Red/Blue flickering stimulus. A casual glance at the figure indicates interesting differences among the categories. The first window showed high entropy values widespread over multiple cortical regions across all participants including both types of patients. This result is in line with our earlier hypothesis, according to which, against a flickering stimulus, a healthy brain would be associated with a high entropy value representing a normal, non-deterministic, and possibly non-linear dynamical state with higher degree of disorder; on the other hand an epileptic brain would be associated with a much lower entropy value, suggesting a higher degree of orderly dynamics.

In adult controls, frontal and temporal regions, bilaterally, showed consistently higher entropy values throughout the post-stimulus period, and the occipital regions, as expected, showed lower entropies. On the contrary, the unmedicated patient showed a sharp reduction in entropy values at first in the occipital region, which later spread throughout the brain, thereby transforming the brain from a highly disordered state, in dynamical sense, to an ordered one. The medicated patient showed the effect of the stimulus only in the occipital region, as the rest of the regions maintained their high entropy throughout the timeline. The entropy values were even higher than that of control participants elucidating the role of medication. Due to a possible inhibition against spreading of low entropy regions to other parts of brain, the medicated patient behaved more like adult controls, thereby avoiding any cortical hyper-excitation. Age-matched controls represented an intermediate state between an adult control and a patient as the entropy values were intermediate to both the categories in most regions. This implies that as compared to a developing brain, a mature brain is associated with more disorder in terms of neuronal dynamics, thereby allowing higher flexibilities in switching between transient states. This flexibility is particularly helpful in defending against photosensitive epilepsy where the brain shows entrainment behaviour by a flickering stimulus.

### Chromatic sensitivity

**Figure 8:**
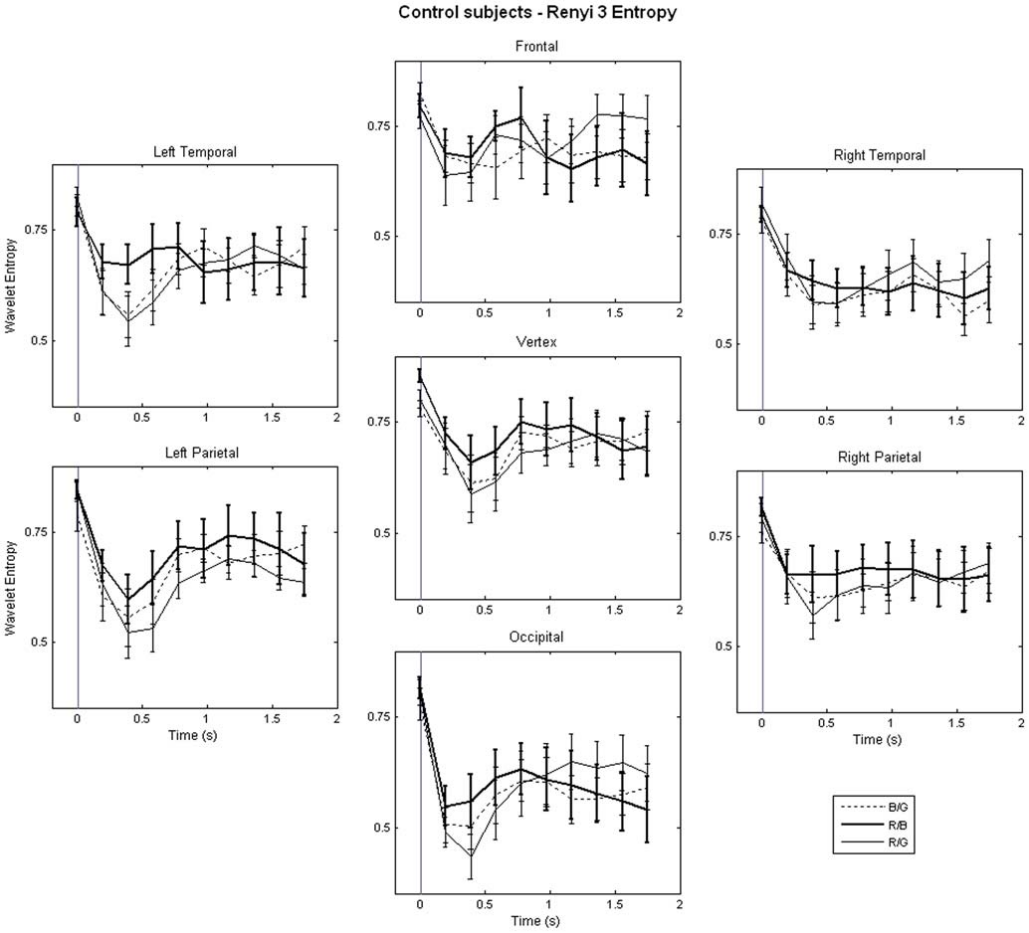
Chromatic Sensitivity Analysis. Temporal evolution of entropy values (mean±s.e.) in seven cortical regions for three different flickering stimuli. Each point represents the center of each window which is 400 ms long. The onset of visual stimulus was indicated by a vertical line. The results for only adult control participants are shown.

One of the primary objectives of our study was to investigate the changes in neuromagnetic brain responses as a factor of different chromatic combinations. Although colour is one of the most common features in any visual stimuli, the effect of combination of colours on PSE has rarely been studied. Earlier, Drew et al. [49] show that pupil constriction was largest for Red/Blue flicker. Since pupil constriction could be considered as a defensive mechanism against PSE, Red/Blue combination is considered the most potent stimulus, amongst other colour combinations, in eliciting PSE. Therefore, we predicted that entropy values for Red/Blue stimulus would be significantly different from two other stimuli. Fig. 8 shows the RE3 values for adult control participants and for all three stimuli. The earliest difference between three stimuli was found at temporal regions at the third time window (200–600 ms) where Red/Blue stimulus produced the largest entropy (*p*<0.05, post-hoc contrast followed by ANOVA). The effect, i.e. higher entropy for Red/Blue than two other conditions, lasted robustly till fifth time window (600–1000 ms) and also in other cortical regions. Interestingly, Red/Blue stimulus elicited lowest entropy at occipital region at the later stages of stimulus processing.

### Effect of medication

The difference in brain responses between the unmedicated patient and the medicated patient attracted a considerable attention in our study, as shown earlier (Figs. 6,7). To begin with, the occipital region of both showed a similar effect of the visual stimulus, leading to low entropy values. This was also evident from the higher ratio (standard error/mean) as shown in Fig. 6. Further, spatiotemporal progression of wavelet entropy took two different routes by the two participants. In the unmedicated patient, the low entropy zone of the occipital region quickly spread to all other regions, ultimately leading to a state of cortical hyper-excitation. However, in medicated patient, the low entropy zone was spatially confined to the occipital region only, thereby the further progression towards a global excitation was inhibited. The high average value of entropy for the medicated patient placed her closer to the control participants in participant classification. We speculated that the anti-epileptic drug (sodium valporate) taken by the medicated patient artificially maintained the general disorderness of the brain, thereby sustaining a very high value of entropy in various regions of the cortex, which possibly helped in preventing the medicated patient from yielding to the visual flickering stimulus.

In summary, the present study presented an application of a recently proposed method of wavelet entropies to the MEG signals for investigating photosensitive epilepsy. The study encompassed various aspects of photosensitivity, the onset of cortical excitation, and its evolution in time-space domain. The study also compared the efficiency of various features based on wavelet entropies. Among the various aspects studied in this paper, a few were based on methodology, while others dealt with the disorder itself. We proposed the use of Renyi Entropy of order 3 as a powerful feature for the analysis of photosensitivity. We also derived a simple index based on temporal variation of wavelet entropy to emphasize the impact of photosensitive epilepsy. Although we robustly separated the unmedicated patient from all other participants including a medicated patient based on entropy values, a possible criticism could be pointed towards the low number of patients. On this aspect, two critical points need to be considered which limited the number of participants: (i) difficulty in getting sufficient number of photo-sensitive patients, (ii) imminent danger associated with the experiment where photosensitive patients are exposed to visual stimuli which could trigger epileptic attack. Therefore, despite the low number of participants, we believed that the adopted methodologies offer novel insight into the dynamics of PSE using MEG data, but further research is needed before confirming the usefulness of the wavelet entropy as a diagnostic marker of PSE.
